# The Hour of Death

**Published:** 1872-06

**Authors:** 


### THE HOUR OF DEATH,

In a natural way, comes to more persons in the neighborhood of five o'clock in the morning, than at any other of the twenty-four; the fewest, about the hour of one in the afternoon.

In the early morning the world is still, the atmosphere heavy with the damps of the night, and the body debilitated, often with the long fast from supper-time, with nothing to rouse the spirits or the circulation.

At about one o'clock in the afternoon the air is most generally fully dried by the sun, has more life, more oxygen in it, hence is more purifying, more invigorating, while the bright daylight itself has an elevating, vitalizing tendency.

These facts should be borne in mind by those who are nurses to the sick, for by extra attentions of various kinds the critical hour might pass, and if so, the patient is more liable to live over for another twenty-four hours.

It is said by observant physicians that each seventh year of life is critical; which means that every seventh year is liable to be fatal; but that if passed over with improved health, it gives a reasonably certain lease of another seven years; for example, the most of those who become consumptive do so about the age of twenty-one, a year sooner or later, but twenty-one is the largest average when the disease becomes decided.

About forty-two, the six times seven, is by far the most critical time of life in women; if that is passed healthfully, they have a good chance of seeing threescore.

It will perhaps be found that a larger number of persons die within a year or two including sixty-three, than at any other specified time between forty-nine and seventy; these things suggest that increased attention should be given to the health at these critical periods.

